# Distribution of Flavonoids and Cyclohexenyl Chalcone Derivatives in Conventional Propagated and *In Vitro*-Derived Field-Grown *Boesenbergia rotunda* (L.) Mansf.

**DOI:** 10.1155/2015/451870

**Published:** 2015-04-07

**Authors:** Boon Chin Tan, Siew Kiat Tan, Sher Ming Wong, Nabeel Ata, Noorsaadah Abd. Rahman, Norzulaani Khalid

**Affiliations:** ^1^Centre for Research in Biotechnology for Agriculture, University of Malaya, 50603 Kuala Lumpur, Malaysia; ^2^Institute of Biological Sciences, Faculty of Science, University of Malaya, 50603 Kuala Lumpur, Malaysia; ^3^Department of Chemistry, Faculty of Science, University of Malaya, 50603 Kuala Lumpur, Malaysia

## Abstract

The distribution patterns of flavonoids and cyclohexenyl chalcone derivatives in conventional propagated (CP) and *in vitro*-derived (CPA) field-grown plants of an important medicinal ginger, *Boesenbergia rotunda*, are described. A total of eight compounds were extracted from six organs (rootlet, rhizome, shoot base, maroon stem, stalk, and leaf) of the CP and CPA plants. Five major chromatographic peaks, namely, alpinetin, pinocembrin, pinostrobin, 4-hydroxypanduratin A, and panduratin A, were consistently observed by high performance liquid chromatography. Nonaerial organs had higher levels of flavonoids than the aerial ones for all types of samples. Among the compounds detected, pinostrobin and 4-hydroxypanduratin A were the most abundant flavonoid and cyclohexenyl chalcone derivative, respectively. The distribution and abundance of the bioactive compounds suggested that the shoot base could be more potentially useful for medicinal application than other organs of the plant and may be the site of storage or occurrence of biosynthetic enzymatic activities.

## 1. Introduction

*Boesenbergia rotunda* (L.) Mansf. (*syn. B. pandurata* (Roxb.) Schltr.), known as fingerroot ginger, is an important member of the Zingiberaceae family due to its medicinal properties. It is extensively used in Asia both in traditional medicine and as a spice or condiment in cooking. Its tubers are widely applied locally to tumors, swellings, and wounds and as a treatment for colic disorders such as diarrhoea [1].

*Boesenbergia rotunda* contains numerous beneficial compounds that have great potential for pharmaceutical applications. Bioactive compounds from rhizome extracts have been identified [2, 3] and classified mainly into two major groups, flavanones (e.g., alpinetin, pinostrobin, and pinocembrin) and chalcones (e.g., boesenbergin, cardamonin, panduratin A, and 4-hydroxypanduratin A) [4]. These compounds have antioxidant, antibacterial, antifungal, anti-inflammatory, antitumour, or antituberculosis activities [5]. For example, cyclohexenyl chalcone derivatives (CCDs), such as panduratin A and hydroxypanduratin A, exhibit anti-inflammatory activity that inhibits the production of both nitric oxide and prostaglandin through the suppression of NF-*κβ* activation [6]. These compounds also significantly reduce the expression of matrix metalloproteinase-1 and induce the expression of type 1 procollagen to a much greater extent than epigallocatechin 3-O-gallate, suggesting that panduratin A could be a potential candidate for preventing or treating skin aging induced by UV radiation [7]. Previous studies have also suggested that panduratin A may serve as an effective preventative or therapeutic chemical agent for cancer [8, 9] and may inhibit TNF-*α* and aminopeptidase N activities [10]. Win et al. [11, 12] reported that panduratin D was cytotoxic against pancreatic cancer cells. Interestingly, cyclohexenyl chalcones derived from* B. rotunda* have been reported to possess antidengue properties, which were mainly attributed to their inhibitory action against the NS3 protease of the DEN-2 virus [13].

Flavonoids are a group of heterocyclic organic compounds [14] that have many diverse functions in plants, including defense, pollination, protection from UV radiation, inhibition of auxin transport, and flower coloring [15]. Pinostrobin has been reported to elevate the activity of an antioxidant enzyme and quinone reductase [16], mediate inflammation, and reduce estrogen-induced cell proliferation [3]. Trakoontivakorn et al. [17] reported that pinostrobin, cardamonin, panduratin A, and 4-hydroxypanduratin A possessed antimutagenic activities. Cardamonin is also able to inhibit HIV-1 protease [18] and has analgesic and antipyretic activities [19].

Flavonoids have a great potential for industrial applications due to their bioactive properties. Despite the potential of these compounds, the limited availability in nature continues to be a significant challenge. Although chemical synthesis is available for some flavonoid compounds, the use of harsh or toxic chemical solvents has limited the synthesis of these high value compounds [20]. Therefore, it is vital to ensure continuous supply of plant source in order to meet the commercial demand.* B. rotunda* is traditionally propagated by vegetative techniques using a rhizome segment [21]. However, this method is slow, time-consuming, and not economically viable as the collection of rhizome for industrial applications could limit the starting material for propagation. Thus, it is essential to develop* in vitro* propagation method for obtaining sustainable, optimized sources of plant-derived bioactive compounds [22]. The morphogenic potential and regenerative capacity of* B. rotunda* from* in vitro* cell cultures have been reported [23], but a detailed analysis of the productive competency and biosynthetic pathway of flavonoids has remained elusive. We have thus examined and compared the production of various flavonoids and chalcones produced in conventional propagated (CP) and* in vitro*-derived (CPA) field-grown plants of* B. rotunda*. The highest yields of bioactive compounds obtained from different organs of* B. rotunda* can thus be identified and exploited for medicinal use.

## 2. Materials and Methods

### 2.1. Plant Materials

**Figure 1:**
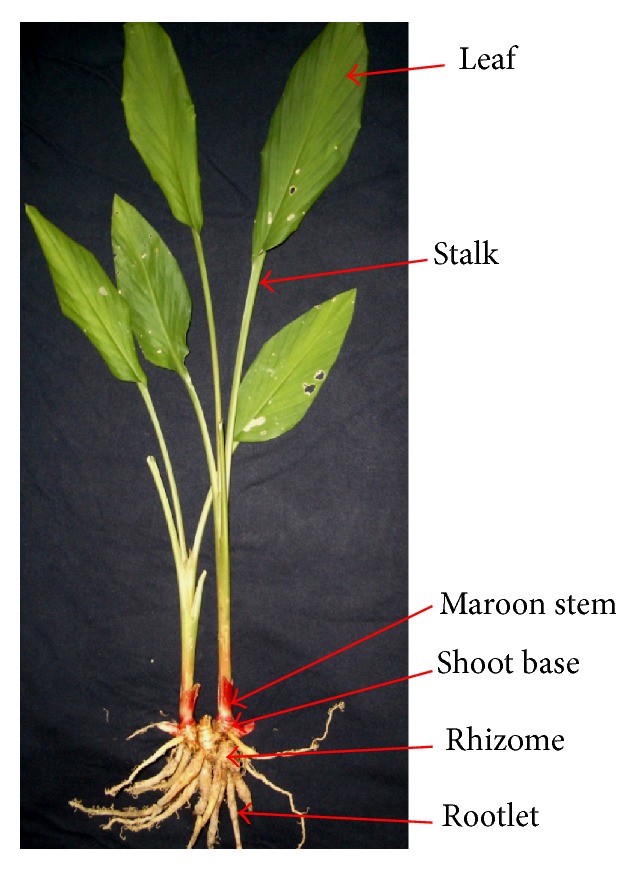
Different segments of* Boesenbergia rotunda *L. (Mansf.) for conventionally propagated and* in vitro*-derived field-grown plants.

Fresh yellow rhizomes of* B. rotunda* obtained from a local farm near Kuala Lumpur, Malaysia, were thoroughly cleaned by rinsing with tap water. The cleaned rhizomes were air-dried before being placed on a layer of clean cotton to allow shoots to sprout to at least 1-2 cm in length. The shoots were surface-sterilized and used as explants to initiate shoots according to the protocol of Tan et al. [23]. To analyze the metabolic profiles in different tissues of* B. rotunda*, CPA of 2-3 cm in height and CP plants were cleaned and separated into six organs: rootlets, rhizomes, shoot-base rhizomes, maroon stems, leaf stalks, and leaves (Figure 1). All organs were thinly sliced, air-dried, and ground into a fine powder using a blender. This powder was stored at −80°C until use.

### 2.2. Extraction and Purification of Bioactive Compounds

Bioactive compounds were extracted by soaking the powders in methanol overnight. The methanolic extracts obtained by filtration were evaporated in vacuum at 35°C, and the resultant slurry was partitioned with equal volumes of ethyl acetate (EA) and water. Partitioning was necessary to remove excessive polar compounds in the extracts. The EA fraction was vacuum-dried, and the mass of the crude extract was recorded. The crude extract was dissolved in methanol at a ratio of 1 mg to 30–60 *μ*L methanol and subsequently filtered through a 0.45 *μ*m PTFE filter (German Acrodisc 13 CR) prior to analysis by high performance liquid chromatography (HPLC).

### 2.3. HPLC Analysis

An injection volume of 50 *μ*L was applied for each sample, and the eluent was monitored at 285 and 330 nm in an HPLC system (Perkin Elmer) equipped with a semipreparative column (Chromolith SemiPrep RP-18 endcapped 100–10 mm column, pore size 2 *μ*m to 13 nm (Merck, Germany), flow rate: 1.5 mL/min), Perkin Elmer Series 200 Pump, diode array detector, and Rheodyne 7725i manual sampler controlled by TotalChrom Navigator Series 200 Workstation software. The solvent system was 80% water-phosphoric acid and 20% acetonitrile for 0.5 min, which was subsequently mixed using a linear gradient starting with 80% phosphoric acid. The gradient was gradually decreasing to 70% (over 5 min) and held for 7 min and then to 50% over 20 min and held for 4 min and finally to 20% over 14 min. Each run was 50.5 min. A total of five runs were conducted for each sample, and peak areas with less than 5% standard deviation were recorded. The compounds were quantified by comparing the absorbance to our previously identified external standards [13]. Naringenin was added to the samples as an internal standard.

### 2.4. Statistical Analysis

The experiments were conducted with five independent replicates and expressed as percentages. The data were analyzed statistically by analysis of variance (ANOVA) followed by Duncan's multiple-range test at a significance level of *P* < 0.05.

## 3. Results and Discussion

The distribution of selected bioactive compounds in CP and CPA field-grown plants was investigated in order to evaluate selected biochemical contents and to understand their site of biosynthesis with the aim of producing higher accumulation of the targeted compounds through genetic manipulation in the future. In this study, rootlets, rhizomes, and shoot bases were considered as nonaerial organs of CP and CPA field-grown plants, whereas leaves, stalk, and maroon stem were classified as aerial organs (Figure 1).

**Figure 2:**
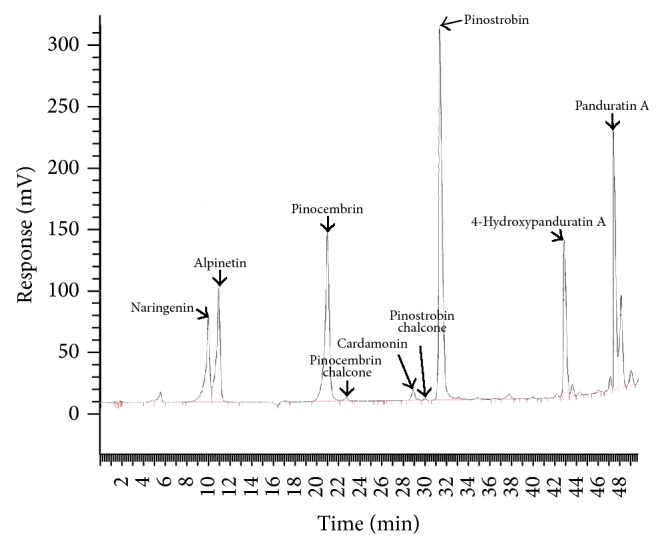
Compounds isolated from* Boesenbergia rotunda*.

**Figure 3:**
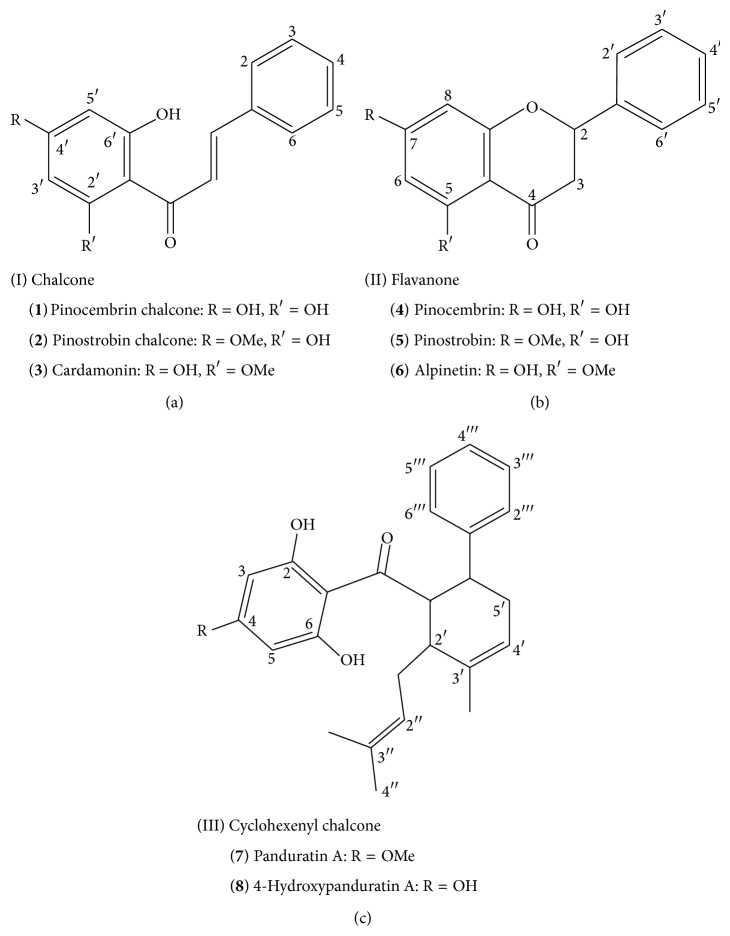
HPLC chromatogram of extract from the shoot base of* Boesenbergia rotunda.*

Eight compounds, namely, alpinetin, pinocembrin chalcone, pinostrobin chalcone, pinostrobin, pinocembrin, cardamonin, 4-hydroxypanduratin A, and panduratin A, were tracked using HPLC throughout retention times between 10 and 46 min (Figures 2 and 3). Of these, alpinetin, pinocembrin, pinostrobin, 4-hydroxypanduratin A, and panduratin A were consistently observed in extracts from nonaerial and aerial organs throughout the analysis.

The nonaerial organs of* B. rotunda* were richer in flavonoids than the aerial organs in both CP and CPA plants (Table 1). Pinostrobin was predominantly present in the nonaerial organs of CP and CPA plants, followed by pinocembrin and alpinetin. We also found considerable differences in pinostrobin levels in different organs of the CP and CPA plants. The pinostrobin contents were about 12% higher in the nonaerial organs of the CPA plants compared to CP plants. The quantity of pinocembrin in the nonaerial organs, except rhizome, varied significantly between the CP and CPA plants. Pinocembrin levels detected in the nonaerial organs were 3% higher in the CPA than in the CP plants. The highest concentration of pinocembrin was detected in the rhizome of CPA plants and shoot bases of CP plants.

In addition, levels of alpinetin in the CP and CPA plants were significantly higher (*P* < 0.05) in the nonaerial organs compared to the aerial organs. Only low levels of cyclohexenyl chalcone derivatives were detected for both types of samples. These derivatives included 4-hydroxypanduratin A, panduratin A, cardamonin, pinostrobin chalcone, and pinocembrin chalcone. 4-Hydroxypanduratin A was more abundant than panduratin A in the CP and CPA plants, whereas minimal amounts of pinocembrin chalcone were detected in either type of sample.

The aerial organs generally had higher levels of the eight selected compounds in the CP plants than in the CPA (Table 1). In general, the aerial organs contained much less pinostrobin than the nonaerial organs, yet considerable amounts of pinostrobin were detected in these organs, with the highest amount (2020.1 *μ*g g^−1^) in the maroon stem of CP plants. In contrast, less pinostrobin than pinocembrin and alpinetin was detected in the CPA. Only a small amount of pinocembrin chalcone (0.8–3.3 *μ*g g^−1^) was detected in the stalks of CP and CPA plants and none in the leaves, similar to the nonaerial organs.

**Figure 4:**
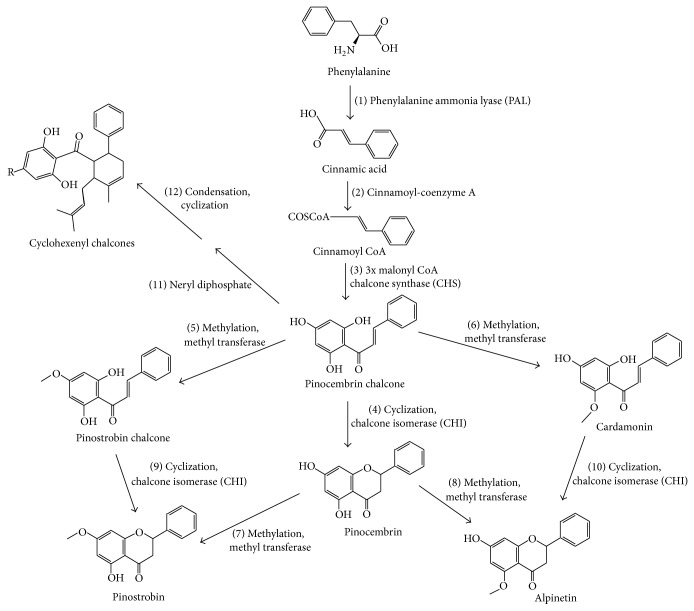
Biosynthetic pathway suggested for the analyzed flavonoids and chalcones.

Based on our findings, we suggest the biosynthesis of selected flavonoids in* B. rotunda* by referring to the previously established pathways (Figure 4) [24–26]. Phenylalanine would be converted to pinocembrin chalcone, a precursor for subsequent flavonoid formation, via a series of deaminations (steps 1, 2, and 3). Thus, the probability of detecting pinocembrin chalcone in regions other than the sites of its biosynthesis would be small. This observation is substantiated by its absence in the rootlets and leaves of CP plants propagated through rhizome and tissue culture methods. In addition, low amounts of pinocembrin chalcone were detected in the other plant organs tested. Higher concentrations of pinostrobin chalcone and its products were found in the shoot base compared to other organs. The conversion of pinocembrin chalcone to cardamonin through methylation was low as cardamonin may have been converted to alpinetin (steps 6 and 10). In contrast, high accumulation of pinocembrin and pinostrobin was detected, indicating the isomerisation and cyclisation of pinocembrin chalcone to pinocembrin is more favorable than to alpinetin. The presence of pinostrobin chalcone was scarce, suggesting its intermediate role in the conversion of pinocembrin chalcone to pinostrobin (steps 5 and 9). The highest amounts of pinostrobin in all organs of the CP and CPA plants suggested its probability as an end product of the biosynthetic pathway. Unlike pinostrobin, pinocembrin is an intermediate compound that could be methylated to form pinostrobin and alpinetin (steps 7 and 8). In comparison, the amount of CCDs (hydroxypanduratin A and panduratin A) was lower than the other end products (pinostrobin and alpinetin), implying that biosynthesis of pinostrobin is the major process in* B. rotunda*.

In this study, the distribution of the flavonoids and chalcones in organs of* B. rotunda* CP and CPA field-grown plants was profiled, which led to a better understanding of the biosynthetic sites and the possible route of formation of the compounds from their respective precursor molecules. Our study could also be useful as a guide for selecting the best plant tissues for extracting compounds for medicinal uses. Nevertheless, further research is required to confirm or amend the proposed biosynthetic pathway.

## Figures and Tables

**Table 1:** The distribution of eight selected compounds in conventionally propagated and *in vitro*-derived field-grown plants.

Plant type	Part	Organ	Bioactive metabolites (*μ*g g^−1^)							
			Alpinetin	Pinocembrin	Pinostrobin	4-Hydroxypanduratin A	Panduratin A	Pinocembrin chalcone	Cardamonin	Pinostrobin chalcone
CP	Aerial	Rootlet	2659.8^c^	3657.4^c^	10502.6^c^	562.8^c^	392.8^b^	0.0^d^	143.3^cd^	52.5^bc^
		Rhizome	3738.0^b^	4918.2^bc^	11366.3^c^	791.4^b^	428.6^b^	0.0^d^	146.2^cd^	95.9^bc^
		Shoot base	3651.6^b^	7038.1^a^	20966.7^a^	1152.7^a^	576.4^a^	126.6^b^	601.5^b^	59.3^bc^
	Nonaerial	Maroon stem	613.0^d^	824.1^d^	2020.1^d^	48.4^d^	38.8^c^	30.6^cd^	70.6^cd^	407.8^a^
		Stalk	81.1^d^	146.0^d^	491.0^d^	7.35^d^	5.2^c^	0.8^d^	13.4^d^	207.4^b^
		Leaf	106.5^d^	159.9^d^	456.7^d^	7.5^d^	2.5^c^	0.0^d^	4.2^d^	477.9^a^

CPA	Aerial	Rootlet	4023.1^b^	5235.3^b^	16845.4^b^	552.5^c^	530.1^ab^	0.0^d^	262.2^c^	37.8^bc^
		Rhizome	4953.0^a^	6109.4^ab^	17098.5^b^	1124.3^a^	588.7^a^	92.6^bc^	178.8^cd^	14.5^c^
		Shoot base	4075.5^b^	5360.9^b^	21049.4^a^	1197.6^a^	584.9^a^	298.9^a^	1031.5^a^	7.1^c^
	Nonaerial	Maroon stem	280.4^d^	404.7^d^	630.954^d^	39.4^d^	17.4^c^	40.3^cd^	21.3^d^	8.7^c^
		Stalk	28.7^d^	61.0^d^	80.1664^d^	4.0^d^	1.5^c^	3.3^d^	3.8^d^	3.0^c^
		Leaf	27.2^d^	33.8^d^	110.4^d^	2.1^d^	2.0^c^	0.0^d^	4.2^d^	3.9^c^

Different letters indicate significant differences at *P* < 0.05.

## References

[1] Tan E. C., Foo G. T., Wong S. M. (2011). Optimization of two-dimensional gel electrophoresis protocol for *Boesenbergia rotunda in vitro* suspension culture. *Journal of Medicinal Plants Research*.

[2] Jing L. J., Mohamed M., Rahmat A., Bakar M. F. A. (2010). Phytochemicals, antioxidant properties and anticancer investigations of the different parts of several gingers species (*Boesenbergia rotunda*, *Boesenbergia pulchella* varattenuata and *Boesenbergia armeniaca*). *Journal of Medicinal Plants Research*.

[3] Yusuf N. A., Suffian M., Annuar M., Khalid N. (2013). Existence of bioactive flavonoids in rhizomes and plant cell cultures of *Boesenbergia rotunda* (L.) Mansf. Kulturpfl. *Australian Journal of Crop Science*.

[4] Kirana C., Jones G. P., Record I. R., McIntosh G. H. (2007). Anticancer properties of panduratin A isolated from *Boesenbergia pandurata* (Zingiberaceae). *Journal of Natural Medicines*.

[5] Eng-Chong T., Yean-Kee L., Chin-Fei C. (2012). Boesenbergia rotunda: from ethnomedicine to drug discovery. *Evidence-Based Complementary and Alternative Medicine*.

[6] Yun J.-M., Kwon H., Hwang J.-K. (2003). *In vitro* anti-inflammatory activity of panduratin A isolated from *Kaempferia pandurata* in RAW264.7 cells. *Planta Medica*.

[7] Shim J.-S., Kwon Y.-Y., Hwang J.-K. (2008). The effects of panduratin a isolated from *Kaempferia pandurata* on the expression of matrix metalloproteinase-1 and type-1 procollagen in human skin fibroblasts. *Planta Medica*.

[8] Yun J.-M., Kwon H., Mukhtar H., Hwang J.-K. (2005). Induction of apoptosis by panduratin A isolated from *Kaempferia pandurata* in human colon cancer HT-29 cells. *Planta Medica*.

[9] Yun J.-M., Kweon M.-H., Kwon H., Hwang J.-K., Mukhtar H. (2006). Induction of apoptosis and cell cycle arrest by a chalcone panduratin A isolated from *Kaempferia pandurata* in androgen-independent human prostate cancer cells PC3 and DU145. *Carcinogenesis*.

[10] Morikawa T., Funakoshi K., Ninomiya K. (2008). Medicinal foodstuffs. XXXIV. Structures of new prenylchalcones and prenylflavanones with TNF-*α* and aminopeptidase N inhibitory activities from *Boesenbergia rotunda*. *Chemical and Pharmaceutical Bulletin*.

[11] Win N. N., Awale S., Esumi H., Tezuka Y., Kadota S. (2007). Bioactive secondary metabolites from *Boesenbergia pandurata* of Myanmar and their preferential cytotoxicity against human pancreatic cancer PANC-1 cell line in nutrient-deprived medium. *Journal of Natural Products*.

[12] Win N. N., Awale S., Esumi H., Tezuka Y., Kadota S. (2008). Panduratins D-I, novel secondary metabolites from rhizomes of *Boesenbergia pandurata*. *Chemical and Pharmaceutical Bulletin*.

[13] Kiat T. S., Pippen R., Yusof R., Ibrahim H., Khalid N., Rahman N. A. (2006). Inhibitory activity of cyclohexenyl chalcone derivatives and flavonoids of fingerroot, *Boesenbergia rotunda* (L.), towards dengue-2 virus NS3 protease. *Bioorganic and Medicinal Chemistry Letters*.

[14] Cushnie T. P. T., Lamb A. J. (2011). Recent advances in understanding the antibacterial properties of flavonoids. *International Journal of Antimicrobial Agents*.

[15] Buer C. S., Imin N., Djordjevic M. A. (2010). Flavonoids: new roles for old molecules. *Journal of Integrative Plant Biology*.

[16] Fahey J. W., Stephenson K. K. (2002). Pinostrobin from honey and Thai ginger (*Boesenbergia pandurata*): a potent flavonoid inducer of mammalian phase 2 chemoprotective and antioxidant enzymes. *Journal of Agricultural and Food Chemistry*.

[17] Trakoontivakorn G., Nakahara K., Shinmoto H. (2001). Structural analysis of a novel antimutagenic compound, 4-hydroxypanduratin A, and the antimutagenic activity of flavonoids in a Thai spice, fingerroot (*Boesenbergia pandurata* Schult.) against mutagenic heterocyclic amines. *Journal of Agricultural and Food Chemistry*.

[18] Tewtrakul S., Subhadhirasakul S., Puripattanavong J., Panphadung T. (2003). HIV-1 protease inhibitory substances from *Boesenbergia pandurata* Holtt. *Songklanakarin Journal of Science and Technology*.

[19] Panthong A., Tassaneeyakul W., Kanjanapothi D., Tantiwachwuttikul P., Reutrakul V. (1989). Anti-inflammatory activity of 5,7-dimethoxyflavone. *Planta Medica*.

[20] Wang Y., Chen S., Yu O. (2011). Metabolic engineering of flavonoids in plants and microorganisms. *Applied Microbiology and Biotechnology*.

[21] Yusuf N. A., Annuar M. M. S., Khalid N. (2011). Rapid micropropagation of *Boesenbergia rotunda* (L.) Mansf. Kulturpfl. (a valuable medicinal plant) from shoot bud explants. *African Journal of Biotechnology*.

[22] Simões-Gurgel C., Cordeiro L. D. S., de Castro T. C., Callado C. H., Albarello N., Mansur E. (2011). Establishment of anthocyanin-producing cell suspension cultures of *Cleome rosea* Vahl ex DC. (Capparaceae). *Plant Cell, Tissue and Organ Culture*.

[23] Tan S. K., Pippen R., Yusof R., Ibrahim H., Rahman N., Khalid N. (2005). Simple one-medium formulation regeneration of fingerroot [*Boesenbergia rotunda* (L.) Mansf. Kulturpfl.] via somatic embryogenesis. *In Vitro Cellular and Developmental Biology—Plant*.

[24] Dixon R. A., Paiva N. L. (1995). Stress-induced phenylpropanoid metabolism. *Plant Cell*.

[25] Hwang E. I., Kaneko M., Ohnishi Y., Horinouchi S. (2003). Production of plant-specific flavanones by *Escherichia coli* containing an artificial gene cluster. *Applied and Environmental Microbiology*.

[26] Jiang H., Wood K. V., Morgan J. A. (2005). Metabolic engineering of the phenylpropanoid pathway in *Saccharomyces cerevisiae*. *Applied and Environmental Microbiology*.

